# A comparison of international and national references to measure the prevalence of stunting in Pakistani school-age girls

**DOI:** 10.1038/s41598-022-09511-3

**Published:** 2022-04-01

**Authors:** Rizwan Qaisar, Asima Karim

**Affiliations:** 1 Department of Basic Medical Sciences, College of Medicine, University of Sharjah, Sharjah, United Arab Emirates; 2 Department of Physiology & Cell Biology, University of Health Sciences, Lahore, Pakistan

**Keywords:** Health care, Paediatrics, Paediatric research

## Abstract

Epidemiology of stunting in < 5 years old is well characterized; however, its prevalence in adolescence is inconsistent in different geographical locations. We estimated the prevalence of stunting in schoolgirls of Punjab, Pakistan, to standardize local references according to international and national references. In this population-wide cross-sectional study, 10,050 schoolgirls aged 8–16 years from 12 different districts of northern, central, and southern Punjab were analyzed. The prevalence of stunting was calculated by applying Centres for Disease Control and Prevention (CDC) and World Health Organisation (WHO) height-for-age references and the local reference for the study population. We used Cohen’s kappa statistics to analyze the agreement of our data with reference values, and chi-square test was used as the test of trend. Marked overestimation of the prevalence of stunting was observed (22.72% and 17.49% according to CDC and WHO, respectively) in comparison to local reference (4.94%). According to CDC and WHO references, there was an increasing trend of prevalence of stunting with higher age; however, data was comparable across all the age groups when local references were applied. We recommend that the prevalence of stunting in school-age girls should be determined by applying local height references rather than international ones to plan health strategies and treatments in the local population.

## Introduction

Stunting is a collective process of diminished body growth that is predominant in the early years of life, i.e., before 3 years of age, and continues into school-age children^1^. It is a common occurrence in developing countries like Pakistan and is classified according to World Health Organization (WHO) expert committee report as Height-for-age Z-score < − 2 standard deviation (SD) from the median height of WHO reference population^2^. Several factors can account for stunting, such as poor nutrition, poor childcare, adverse environmental and cultural conditions, and recurrent chronic infections^3^. Stunting is also associated with neurocognitive impairment, poor educational achievement, insufficient social progress, increased cardiometabolic disease risk, increased risk of obesity, and adverse pregnancy outcomes in females^4–8^. Recently, new factors, including social identity, prestige, and ego have emerged as critical drivers of human body development. Collectively called as social-economic-political-emotional (SEPE) environment, these factors contribute to human height and other growth parameters. Thus, the individuals with higher SEPE class are taller than those with lower SEPE class, suggesting a role for SEPE in human growth^9,10^.

Gender bias and female discrimination is common practice in Southeast Asia, with preference given to male children over female children^11^. Male children are typically sent to better class schools for formal education and given better quality and/or quantities of food than their female counterparts especially in Southeast Asia^11–13^, contributing to a higher prevalence of stunting in females vs. male children^14^. It is essential to identify stunting in young females as this is associated with problems in future pregnancies and the birth of premature and low birth weight babies^15^. Most of the studies conducted on Pakistani children to assess stunting have targeted the paediatric population less than 5 years of age^16–19^. Data about stunting in school-age children is limited^14,20–24^.

It is essential to characterize the prevalence of stunting in the high-risk paediatric population of Pakistan, so that the underlying factors can be identified. Stunting in children ≤ 5 years of age is assessed by the universally accepted WHO height-for-age Z-score reference^25^. However, no consistent global reference is applied for school-aged children and adolescents. Two internationally accepted references, i.e., Centre for Disease Control (CDC) and WHO^25,26^ for estimating stunting, are recommended in public health research and clinical practice. The prevalence of stunting would vary by applying each of these references due to their different diagnostic criteria. Four studies conducted in Pakistan among the school-age children to determine the height status have used the WHO reference and have obtained variable/conflicting results^14,21–23^. These disagreements can be attributed to the differences in the population and the methodology employed to generate the height cut-offs. Therefore, to correctly categorize height status in Pakistani children, population-specific height cut-offs need to be generated and validated. The recommended two references have not been applied extensively to the Pakistani school-age population to study the similarities and differences in height status. Moreover, the local height cut-offs compared to these international references will help understand the degree of agreement in classifying height status among schoolgirls of Punjab, Pakistan.

The regional differences can contribute to inconsistent data about stunting in Pakistan^14,20–24^. However, data evaluating these regional differences is limited, and no conclusion can be drawn. Highlighting the impact of these regional differences upon the prevalence of stunting will help tackle this issue in school children in a more efficient manner by modifying the area-based programs. Centile curves for height have recently been generated, comparing the percentile values of height with international references^27^, but estimates of height status are unknown in our study population. Moreover, the regional differences were not considered previously.

In this study, we aimed to fill these gaps by determining the degree of agreement of our local height-for-age cut-offs obtained from 8 to 16-year old schoolgirls compared to CDC and WHO references. We estimated height status in Punjab and then dissected our data in northern, central, and southern Punjab. We hypothesized that the height status of our study participants is only slightly comparable with the international references, and there is marked inconsistency between various regions of Punjab.

## Methods

### Study group

Stunting was classified based upon WHO 2007 reference with height ˂ − 2SD from the median height of the reference population^25^ and height ˂ 5th percentile of the reference population according to CDC 2000 reference^26^. The local reference used in the study has been obtained from our dataset of 10,050 schoolgirls, and stunting was classified based upon height ˂ − 2SD from the median height of the reference population. Briefly, 10,050 school-age girls were recruited by using a stratified multi-stage cluster sampling technique^28^. The study participants were 8–16 years of age and were enrolled from 12 districts and 35 public and private schools in semi-urban, urban, and rural Punjab. Punjab province was subdivided into three strata i.e., northern, central, and southern Punjab, due to topographical, geographical, cultural, environmental and population differences. The three regions of Punjab vary markedly in terms of population density; therefore 1355 (13.5%) girls were recruited from northern, 6580 (65.47%) from central and 2115 (21%) from southern Punjab. From each sub-stratum identified, 12 out of 36 districts (geographical division by the Government) were selected as clusters. Districts were stratified using probability proportional to size technique taking into consideration the percentage of adolescent population in each district after obtaining information from Pakistan census bureau^29^. The ethical approval of the study was obtained from The Institutional Ethical Review Committee (vide letter no UHS/ERB/22546/2014) and was conducted at the Department of Physiology & Cell Biology, University of Health Sciences, Lahore, starting in January 2015. This study was conducted in accordance with the Declaration of Helsinki^30^.

### Anthropometric measurements

Anthropometric measurements are non-invasive quantitative measurements of the body. According to CDC anthropometric measurements provide a valuable tool for assessing nutritional status in children and adults^31^. Anthropometric measurements were recorded for everyone by trained personnel following CDC’s recommendations.

### Statistical analysis

All data entry and analysis were performed using SPSS Version 15.0 (SPSS Inc. Chicago IL, United States, 2009). Cohen’s *k* statistic was used to assess the agreement in the classification of stunting between the international references CDC, WHO, and our study population Height-for-age cut-offs. *k* ˂ 0.6 was defined as the poor agreement, 0.6 to ˂ 0.8 as moderate agreement, and ≥ 0.9 as excellent agreement^32^. Chi-square test was used as a test of the trend to compare differences in the prevalence of stunting between the groups under study, *p*-value ≤ 0.05 was considered statistically significant.

### Ethics approval and consent to participate

The study was approved by the institutional ethical review committee of University of Health sciences, Lahore. An informed written consent was obtained from the parents or legal guardians of schoolgirls participated in this study.

## Results

**Figure 1:**
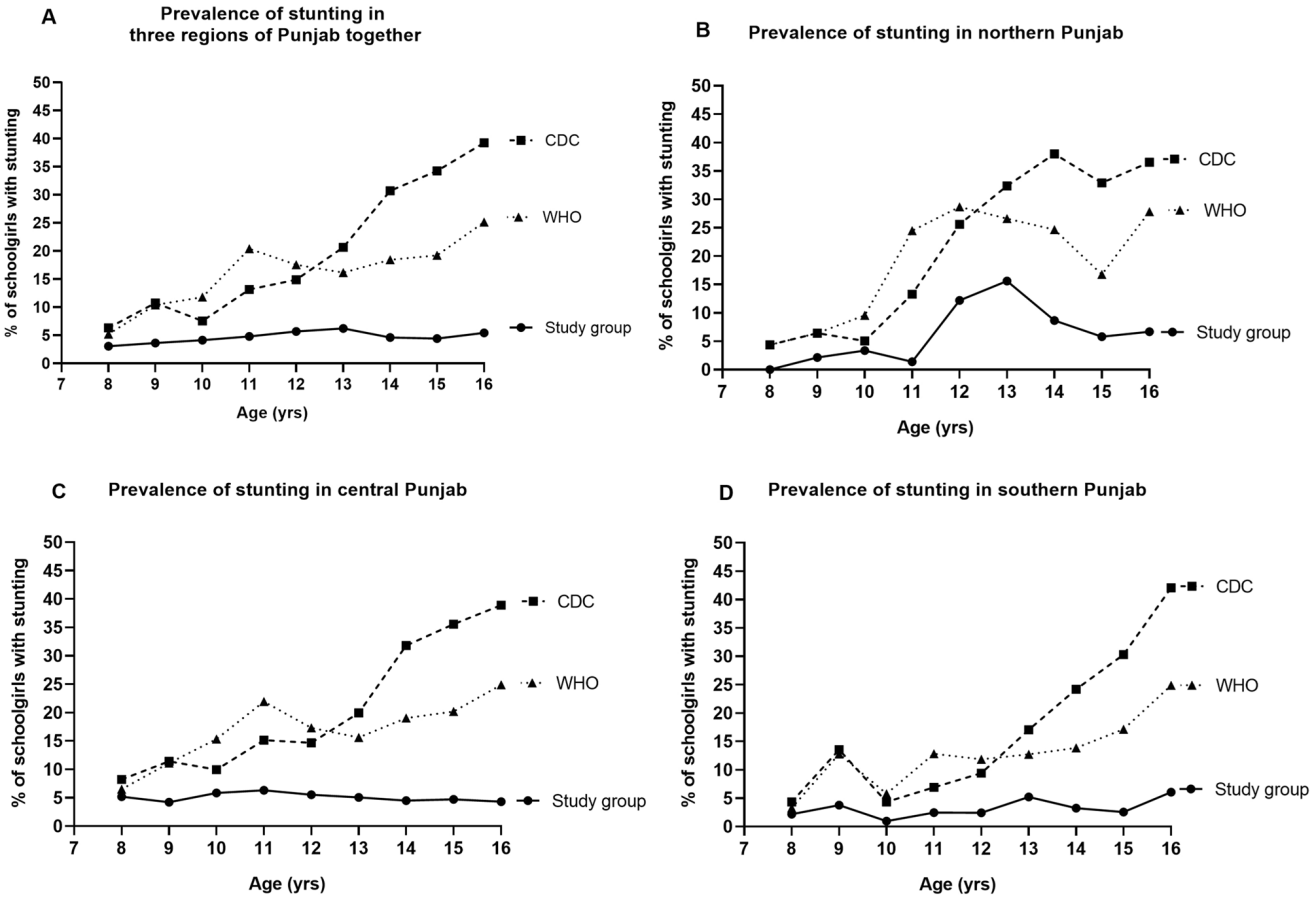
Age-related prevalence of stunting in schoolgirls 8–16 years using the height-for-age references of CDC 2000 (dashed line), WHO 2007 (dotted line), and local reference of study group (solid line) in (**A**) Punjab (n = 10,050); (**B**) northern Punjab (n = 1355); (**C**) central Punjab (n = 6580) and (**D**) southern Punjab (n = 2115).

The mean age of 10,050 schoolgirls included in this study was 12.7 ± 2.29 years (Mean ± SD). The overall prevalence of stunting in the study population using two international references is described in Fig. 1A. Overall, the percentage of girls with normal height in different age groups under study was quite similar (77.28% and 82.51%, respectively). The prevalence of stunted girls in our study cohort was 22.72% and 17.49%, according to CDC and WHO, respectively. The prevalence of stunting was similar for younger girls (8, 9 years) according to CDC and WHO, higher among girls of 10–12 years according to WHO compared to CDC and highest among girls of 13–16 years of age according to CDC compared to WHO reference (Table 1, Fig. 1A). When we applied the local height-for-age cut-offs obtained from our study population, a significantly higher percentage of the girls had standard height (95.06%, *p* ˂ 0.05, Table 1). The overall prevalence of stunting was markedly lower (4.94%, *p* ˂ 0.05, Fig. 1A) in all age groups when using the local reference, compared to CDC and WHO references. However, among 8-year-old girls, the prevalence of stunting was comparable to all the three references applied. A notable finding was the increasing trend of prevalence of stunting with increasing age when CDC and WHO references were applied. However, no such trend was observed when the local cut-offs were applied (Fig. 1A). We compared the height values obtained from our cumulative study cohort with CDC and WHO references and used kappa correlation to assess the degrees of agreement between these references and local references. There was poor agreement between the local reference in comparison with CDC and WHO (*κ* = 0.163, 0.325 respectively) references.

**Table 1:** Prevalence of stunting by applying height-for-age specific cut-offs from CDC, WHO, and the study group in the girls of Punjab; (n = 10,050).

Height status categories	8 y	9 y	10 y	11 y	12 y	13 y	14 y	15 y	16 y	Overall
**CDC reference**										
No. of children	460	606	849	980	1283	1608	1514	1425	1325	10,050
Stunting (%)	6.30	10.73	7.54	13.16	14.89	20.65	30.71	34.25	39.25	22.72
**WHO reference**										
No. of children	460	606	849	980	1283	1608	1514	1425	1325	10,050
Stunting (%)	5.22	10.40	11.78	20.41	17.54	16.17	18.43	19.23	25.13	17.49
**Study group reference**										
No. of children	460	606	849	980	1283	1608	1514	1425	1325	10,050
Stunting (%)	3.04	3.63	4.12	4.80	5.69	6.22	4.62	4.42	5.43	4.94

To investigate the region-specific differences, we sub-categorized the data according to three regions of Punjab, i.e., northern, central, and southern Punjab. The height status of the girls differed across the different subgroups for cut-off values of CDC, WHO, and local standards. In northern Punjab, accumulative estimates for the percentage of girls of all age groups with normal height were variable when the two references were applied (78.52%, 81.18; according to CDC and WHO, respectively) with the highest percentage found when local cut-offs were applied (93.54%, *p* ˂ 0.05; Table 2, Fig. 1B). Overall estimates of stunted girls in northern Punjab were in agreement when these two cut-offs were applied (21.48% and 18.82% according to CDC and WHO references, respectively). When the individual age groups were assessed, a similar percentage of girls were stunted for ages 8 and 9 years according to CDC and WHO references (4.38% and 6.43% for CDC and WHO respectively). Girls of ages 10–12 years were more stunted according to WHO (9.55%, 24.48% and 28.66% respectively) as compared to CDC reference (5.06%, 13.29% and 25.61% respectively), 13–16-year-old girls had a higher prevalence of stunting according to CDC (32.37%, 38%, 32.90% and 36.52% respectively) as compared to WHO reference [26.59%, 24.67%, 16.77% and 27.83% respectively (Table 2. Figure 1B)]. Overall, stunting prevalence in northern Punjab had an agreement across the studied age groups when the two international references were applied. However, the prevalence of stunting was significantly lower with local cut-offs (6.46%, *p* ˂ 0.05) in comparison to the two international references, with the highest prevalence among the 12 and 13-year-old-girls (12.20%, 15.61% girls stunted respectively).

**Table 2:** Prevalence of stunting by applying height-for-age specific cut-offs from the CDC, WHO, and the study group in the girls of northern Punjab; (n = 1355).

Height status categories	8 y	9 y	10 y	11 y	12 y	13 y	14 y	15 y	16 y	Overall
**CDC reference**										
No. of children	137	140	178	143	164	173	150	155	115	1355
Stunting (%)	4.38	6.43	5.06	13.29	25.61	32.37	38.00	32.90	36.52	21.48
**WHO reference**										
No. of children	137	140	178	143	164	173	150	155	115	1355
Stunting (%)	4.38	6.43	9.55	24.48	28.66	26.59	24.67	16.77	27.83	18.82
**Study group reference**										
No. of children	137	140	178	143	164	173	150	155	115	1355
Stunting (%)	0.00	2.14	3.37	1.40	12.20	15.61	8.67	5.81	6.67	6.46

Estimates of girls with standard height in the central Punjab region were comparable to two international references applied (75.71%, 81.53% according to CDC and WHO references, respectively), and the highest prevalence of girls with standard height was found with the local cut-offs (94.95%, *p* ˂ 0.05, Table 3, Fig. 1C). Overall estimates of stunting were very high when the two references were applied, with the highest prevalence obtained with CDC reference (24.29%, 18.47% according to CDC and WHO respectively, Fig. 1C). When CDC, WHO and local references were assessed for all the age groups individually, girls of 8 and 9 years had a similar prevalence of stunting, while a higher prevalence was found in 10–12-year-old girls according to WHO, compared to CDC and local criteria. Stunting was more prevalent in 13–16 years old girls according to CDC as compared to WHO (Fig. 1C). Markedly low estimates of stunting were observed in all the age groups when local cut-offs were applied (5.05%, *p* ˂ 0.05) in comparison to CDC and WHO. Generally, among the girls of central Punjab, a higher prevalence of stunting was observed when applying the CDC and WHO references with increasing age. However, when local cut-offs were applied, the estimates for stunting among the girls across all the age groups analyzed was comparable.

**Table 3:** Prevalence of stunting by applying height-for-age specific cut-offs from the CDC, WHO, and the study group in the girls of central Punjab; (n = 6580).

Height status categories	8 y	9 y	10 y	11 y	12 y	13 y	14 y	15 y	16 y	Overall
**CDC reference**										
No. of children	231	333	463	634	832	1089	1025	996	977	6580
Stunting (%)	8.23	11.41	9.94	15.14	14.66	19.93	31.80	35.54	38.89	24.29
**WHO reference**										
No. of children	231	333	463	634	832	1089	1025	996	977	6580
Stunting (%)	6.49	11.11	15.33	21.92	17.31	15.61	19.02	20.18	24.87	18.47
**Study group reference**										
No. of children	231	333	463	634	832	1089	1025	996	977	6580
Stunting (%)	5.19	4.20	5.83	6.31	5.53	5.05	4.49	4.72	4.31	5.05

In southern Punjab, 81.37% and 86.38% of girls had standard height according to CDC and WHO, respectively (Table 4, Fig. 1D). However, the highest percentage of girls (96.82%) with normal height was obtained when local cut-offs of height were applied. Accumulative prevalence of stunting was very high according to the two references, with the highest prevalence reported when CDC reference applied (18.63%, 13.62% according to CDC and WHO, respectively; Fig. 1D). Next, we investigated the prevalence of stunting among the girls of each age group. In the younger age groups, i.e., 8–10 years’ data according to CDC and WHO was comparable. In 11 and 12-year-old girls of southern Punjab, a lower prevalence of stunting was seen with CDC compared to WHO, and from ages 13–16 years, a higher prevalence was found with the CDC reference (Fig. 1D). When the local cut-offs were employed, a significantly lower prevalence of stunting was found in the girls of southern Punjab (3.18%; *p* ˂ 0.05) compared to CDC and WHO in all the age groups overall with comparable values seen in the individual age groups. When CDC and WHO references were applied, an increasing trend in stunting was observed with increasing age in the study participants (Fig. 1D).

**Table 4:** Prevalence of stunting by applying height-for-age specific cut-offs from the CDC, WHO, and the study group in the girls of southern Punjab; (n = 2115).

Height status categories	8 y	9 y	10 y	11 y	12 y	13 y	14 y	15 y	16 y	Overall
**CDC reference**										
No. of children	92	133	208	203	287	346	339	274	233	2115
Stunting (%)	4.35	13.53	4.33	6.90	9.41	17.05	24.19	30.29	42.06	18.63
**WHO reference**										
No. of children	92	133	208	203	287	346	339	274	233	2115
Stunting (%)	3.26	12.78	5.77	12.81	11.85	12.72	13.86	17.15	24.89	13.62
**Study group reference**										
No. of children	92	133	208	203	287	346	339	274	233	2115
Stunting (%)	2.17	3.76	0.96	2.46	2.44	5.20	3.24	2.55	6.06	3.18

**Figure 2:**
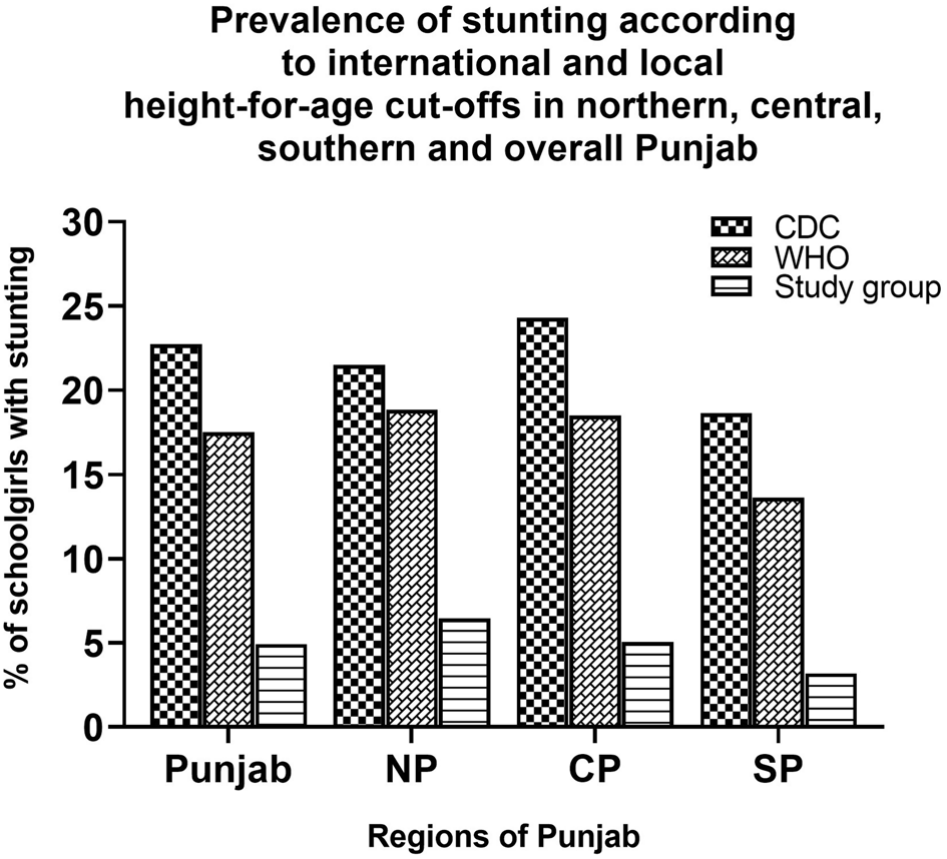
Prevalence of stunting in schoolgirls 8–16 years according to Height-for-age cut-off values of international and local references in Punjab and its three regions.

When the three regions of Punjab were compared for height status by applying local height-for-age cut-offs, the highest prevalence of stunting was observed among the girls belonging to northern Punjab and the lowest prevalence among the girls of southern Punjab (Fig. 2). The CDC reference shows consistently higher rates of stunting.

## Discussion

Our data confirm our hypothesis that significant differences exist in the physical parameters of the study cohort when compared to internationally recognized references. Additionally, we also report a region-specific variation in different parts of Punjab. We found a high prevalence of stunting, with statistically significant discrepancies, when using two international height references. Prevalence of stunting was highest with CDC compared to WHO reference and local reference. Overall, the two international references for height-for-age showed a moderate degree of agreement among themselves when they were applied to estimate stunting (Fig. 1A). However, they showed a poor agreement when CDC and WHO references, compared with the local height-for-age cut-offs. These findings highlight the importance of choosing the appropriate references to estimate the height status of children and adolescents to determine stunting, as it can significantly impact the strategies implemented by policymakers to address this abnormality. An appropriate assessment of stunting can also affect the decision of paediatricians to treat such a condition as currently, there is growing evidence that catch-up growth is possible until 15 years of age^33^.

We found an overestimation of the prevalence of stunting when WHO standards were applied to our Pakistani school-age children^14,21^. A similar trend was observed when CDC and WHO references were applied to determine height status in our study population. There was an increasing trend of stunting with increasing ages in the schoolgirls when CDC and WHO references were used; however, prevalence of stunting was comparable across all the age groups when the local reference cut-offs for the study population were applied. We found approximately 5% stunting in all schoolgirls of Punjab, partly agreeing with a previous study where they found 8% of children were stunted^21^. However, it is in contrast to another study which shows stunting prevalence of 16% (6–12 years)^14^. Moreover, conflicting results with stunting of 16.7% & 14.3% [5–14 years; according to National Health Survey of Pakistan (NHSP) and Karachi surveys respectively]^34^, 15.2% (5–14 years)^20^, 45.2% (4–12 years), 36% (6–12 years)^23^ and 35% (5–10 years)^24^ were found among school children from various regions of the country. However, those studies were conducted on a younger population (range = 5–14 years) with smaller sample sizes (range n = 200–2072) than this study (n = 10,050). Further, only the WHO reference was applied in those studies. When WHO references were applied, the overall prevalence of stunting was 17.49%, which is largely in agreement with the stunting prevalence reported in previous studies as 16%^14^, 15.2%^20^, 16.7% and 14.3%^34^ from Pakistan. At the time of writing, there is no published literature where local reference cut-off for height is applied, rendering it impossible to compare these findings with previous studies. The prevalence of stunting by applying international standards in a few studies from Pakistan show higher values and results inconsistent with these findings^14,20,23,24,34^.

Significant differences were noticed in the estimates of stunting among different regions of Punjab. The highest prevalence of stunting was found among the schoolgirls from northern Punjab and the lowest prevalence in southern Punjab. Interestingly, there was very high stunting estimate among the age groups 12 and 13 years, respectively. The differences in socioeconomic status, maternal education status, provision of health facilities, heterogeneous nature of the population, diverse cultural practices, variable food habits, level of child’s growth and development associated with this chronic malnutrition condition may account for these region-specific differences^35^. Recently, an association between SEPE environment and skeletal growth of the human body has been reported. Thus, the individuals with a higher SEPE class receive positive stimulation and grow taller, while the individuals with a lower SEPE class receive negative stimulation and stunted growth^10^. In addition to SEPE class, the contribution of several other factors, such as fear, violence, inequality, biosocial stress, to human growth has also been recognized^9^. Therefore, it is possible that the height and growth differences in our cohort than international standards care partly due to SEPE and other recently identified factors.

This study emphasizes the importance of comparing international references to the local study population for estimating stunting. The need to generate population-specific height references has been highlighted because of variations in height patterns among pre-adolescent and adolescent children, especially 7–18 years of age^36^. Accordingly, it is not justifiable to use a single reference throughout the globe to estimate height status, especially for Asians^36^. There are huge differences between the height status of adolescents belonging to different populations, suggesting variations in growth patterns depending upon the genetic and environmental factors between these populations^37^. Moreover, for the authenticity of the population-specific cut-offs, longitudinal studies with comprehensive application of references are required. There is very limited data available to assess the adult health status after applying local references. Although there exists a unanimous consensus for the application of standards to estimate stunting in the younger age groups i.e., till 5 years of age, height-for-age reference values have not been specified for the young adolescent group, thereby rendering it impossible to obtain consistent results regarding the prevalence of stunting among school age children.

This study has certain limitations. It is a cross-sectional study, and stunting prevalence was estimated at a single time point. This study only investigated girls but not the boys. Third, the study participants only included the schoolgirls belonging to the province of Punjab. This study needs to be expanded to encompass data from all four provinces of Pakistan.

## Conclusions

We report marked differences in the estimation of stunting prevalence among 8–16-year-old schoolgirls of Punjab after applying CDC, WHO, and local height references. Region-wise differences were also discovered in the prevalence of stunting across all the age groups. The CDC and WHO references showed a significant overestimation of stunting in the study cohort compared to the local reference. There was an increasing trend of stunting prevalence with the increasing age of the girls. The data highlights the importance of generating population-specific height references to determine and monitor stunting in adolescents. The estimation of stunting should be consistent throughout the population to implement appropriate health policies to combat this nutritional problem in our school-age population.

## Data Availability

Data is available from the corresponding author on reasonable request.

## Abbreviations

CDC: Centres for Disease Control and Prevention
IREC: Institutional Ethical Review Committee
NHANES: National Health and Nutrition Examination Survey
WHO: World Health Organization
